# Bi_2_Se_3_/C Nanocomposite as a New Sodium-Ion Battery Anode Material

**DOI:** 10.1007/s40820-018-0201-9

**Published:** 2018-05-03

**Authors:** Lixin Xie, Ze Yang, Jingying Sun, Haiqing Zhou, Xiaowei Chi, Hailong Chen, Andy X. Li, Yan Yao, Shuo Chen

**Affiliations:** 1 Department of Physics and TcSUH, University of Houston, Houston, TX 77204 USA; 2 Department of Electrical and Computer Engineering and Materials Science and Engineering Program, University of Houston, Houston, TX 77204 USA; 3 The Woodruff School of Mechanical Engineering, Georgia Institute of Technology, Atlanta, GA 30332 USA; 4 Clements High School, 4200 Elkins Dr, Sugar Land, TX 77479 USA

**Keywords:** Bi_2_Se_3_, Sodium-ion battery, High-energy ball milling, Sodium storage mechanism

## Abstract

Bi_2_Se_3_ was studied as a novel sodium-ion battery anode material because of its high theoretical capacity and high intrinsic conductivity. Integrated with carbon, Bi_2_Se_3_/C composite shows excellent cyclic performance and rate capability. For instance, the Bi_2_Se_3_/C anode delivers an initial capacity of 527 mAh g^−1^ at 0.1 A g^−1^ and maintains 89% of this capacity over 100 cycles. The phase change and sodium storage mechanism are also carefully investigated.

**Electronic supplementary material:**

The online version of this article (10.1007/s40820-018-0201-9) contains supplementary material, which is available to authorized users.

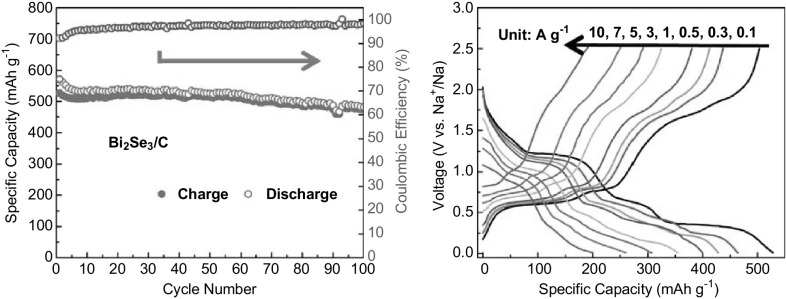

## Highlights

- Bi_2_Se_3_ was investigated as a novel sodium-ion battery anode material.
- Sodiation/desodiation mechanism of Bi_2_Se_3_ has been carefully investigated.
- Bi_2_Se_3_/C electrode demonstrates high cycling stability.

## Introduction

Sodium-ion batteries (SIBs) have recently regained extensive research interest as alternatives to lithium-ion batteries (LIBs) for energy storage owing to the low cost and abundance of Na [1–5]. The lack of high energy density anode materials has impeded the progress of SIBs for a long time [6]. Developing suitable anode materials for SIBs with both high capacity and long cycle life is highly desired. Among anode materials, alloying-type materials [7] have attracted much attention. For example, Sn, Sb, and Bi can reversibly alloy with Na^+^ and provide high theoretical gravimetric capacities (> 300 mAh g^−1^), which far exceed the capacities of carbonaceous materials and Ti-based materials. The accompanying challenge for alloying-type materials is the large volume expansion when alloying with Na^+^. Bi displays a relatively small volume expansion (ca. 250% expansion from Bi to Na_3_Bi), compared to Sn (ca. 420% expansion from Sn to Na_3.75_Sn) and Sb (ca. 293% expansion from Sb to Na_3_Sb) [8], which is beneficial for a stable anode [9]. The voltage plateau is also an important criterion in evaluating an electrode material. A low operating voltage for anode materials can endow a cell with a high operation voltage. However, Na plating, dendrite formation, and electrolyte decomposition occur on the anode side when the discharge voltage approaches 0 V, as is often the case for hard carbon anodes [10–12]. The plateaus of Bi between 0.3 and 0.9 V versus Na^+^/Na are favorable for maintaining a high operation voltage and avoiding the aforementioned detrimental effects [13, 14].

Sulfides and selenides have been actively investigated because their conversion reactions offer high capacities for ion storage [15–18]. Recently, the Bi-based compound Bi_2_S_3_ has been synthesized and displayed a high Na storage capacity [19, 20]. However, the rate capacity was unsatisfactory, limited by the low intrinsic conductivity of sulfides [15]. Bi_2_Se_3_ displays an electrical conductivity two orders of magnitude higher than that of Bi_2_S_3_ [21], which can improve the electron transport. In addition, the shuttle effect is relieved for selenides compared to sulfides [22]. Moreover, Bi_2_Se_3_ has a high density of 7.47 g cm^−3^ [21], permitting the opportunity to fabricate small-sized devices with high volumetric capacities (theoretically 3667 mAh cm^−3^). Bi_2_Se_3_ has been applied in LIBs and exhibited excellent electrochemical storage ability for Li^+^. Several Bi_2_Se_3_ nanostructures, such as nanosheets and microrods, have been designed for Li^+^ storage [23, 24]. Furthermore, high free electron densities can effectively improve the rate capability; thus, doping strategies have been employed to create S-doped and In-doped Bi_2_Se_3_ [25–27]. Despite the good electrochemical performance in Li^+^ storage, Bi_2_Se_3_ has not been reported as an anode material for SIBs.

Downsizing the bulk material to nanoscale and integrating carbon with it can improve the electrochemical performance, including the rate capability and cyclability, by the shorter diffusion distances, more abundant reaction sites on the large surface area, and additional space for expansion [28–31]. Carbon can stabilize the nanomaterial and provide an interconnected network for electron transport as well, and the voids in the carbon can accommodate volume expansion and allow permeation of the electrolyte for fast Na^+^ transport [32–34].

In our study, a simple high-energy ball milling (HEBM) method was adopted to synthesize Bi_2_Se_3_ and Bi_2_Se_3_/C nanocomposite. The Bi_2_Se_3_/C nanocomposite delivers an initial reversible capacity of 527 mAh g^−1^ at 0.1 A g^−1^ with 89% retention over 100 cycles. The phase changes during cycling were investigated by ex situ X-ray diffraction (XRD) to reveal the Na storage mechanism. The rational material design combined with effective synthetic protocol is important and this work is expected to shed light on future work on developing excellent anode materials for SIBs.

## Experimental

### Synthesis Process

The synthesis of Bi_2_Se_3_ and Bi_2_Se_3_/C was performed by HEBM. Bi (Alfa Aesar, 99.999%) and Se (Alfa Aesar, 99.999%) in a molar ratio of 2:3 were sealed in an Ar-filled stainless steel jar and then ball milled for 10 h at 1200 rpm (Spex 8000 M) to form phase-pure Bi_2_Se_3_ powder. Graphite powders were milled for 48 h beforehand. Then, the milled graphite was added to Bi_2_Se_3_ powders in the weight ratio of 2:8 and ball milled for another 6 h to form the carbon-integrated Bi_2_Se_3_ nanocomposite.

### Material Characterization

The phases were investigated by XRD on a Rigaku SmartLab diffractometer with a Cu Kα source at the scan rate of 5 deg. min^−1^. The morphology was studied under scanning electron microscopy (SEM, LEO 1525). The nanostructures and the diffraction patterns were characterized by transmission electron microscopy (TEM, JEOL 2010F, operated under 200 kV). The elemental mapping was collected by energy-dispersive X-ray spectroscopy (EDS) (attached to the TEM). X-ray photoelectron spectroscopy (XPS) measurements were performed on a PHI Quantera XPS instrument. To confirm the carbon content, the samples were heated at 10 °C min^−1^ from room temperature to 600 °C in thermogravimetric analysis (TGA, Q500).

### Electrochemical Measurements

Coin cells (CR 2025) with Bi_2_Se_3_ or Bi_2_Se_3_/C as the active material were assembled for battery tests. A slurry was made by mixing 70 wt% active material, 20 wt% carbon black, and 10 wt% polyacrylic acid (PAA) and then coated on a Cu foil to form the working electrodes, followed by drying at 60 °C under vacuum overnight. To prepare the electrolyte, 1 mol L^−1^ NaClO_4_ was dissolved in propylene carbonate/ethylene carbonate (1:1 in volume) with 5 wt% fluoroethylene carbonate (FEC) as an additive. The loading of the active materials was 1.4 ± 0.2 mg cm^−2^ for the Bi_2_Se_3_/C electrode and 1.5 ± 0.3 mg cm^−2^ for the Bi_2_Se_3_ electrode. Homemade Na lumps and glass fibers were applied as the reference/counter electrodes and the separators, respectively. The electrochemical measurements of the cells were performed galvanostatically between 0.01 and 2.5 V versus Na/Na^+^ on a Land CT2001A battery tester. Cyclic voltammetry (CV) curves were swept at 0.1 mV s^−1^ on a BioLogic SP-200 electrochemical workstation. Electrochemical impedance spectroscopy (EIS) was measured from 100 kHz to 100 mHz with a voltage amplitude of 5 mV.

## Results and Discussion

**Fig. 1:**
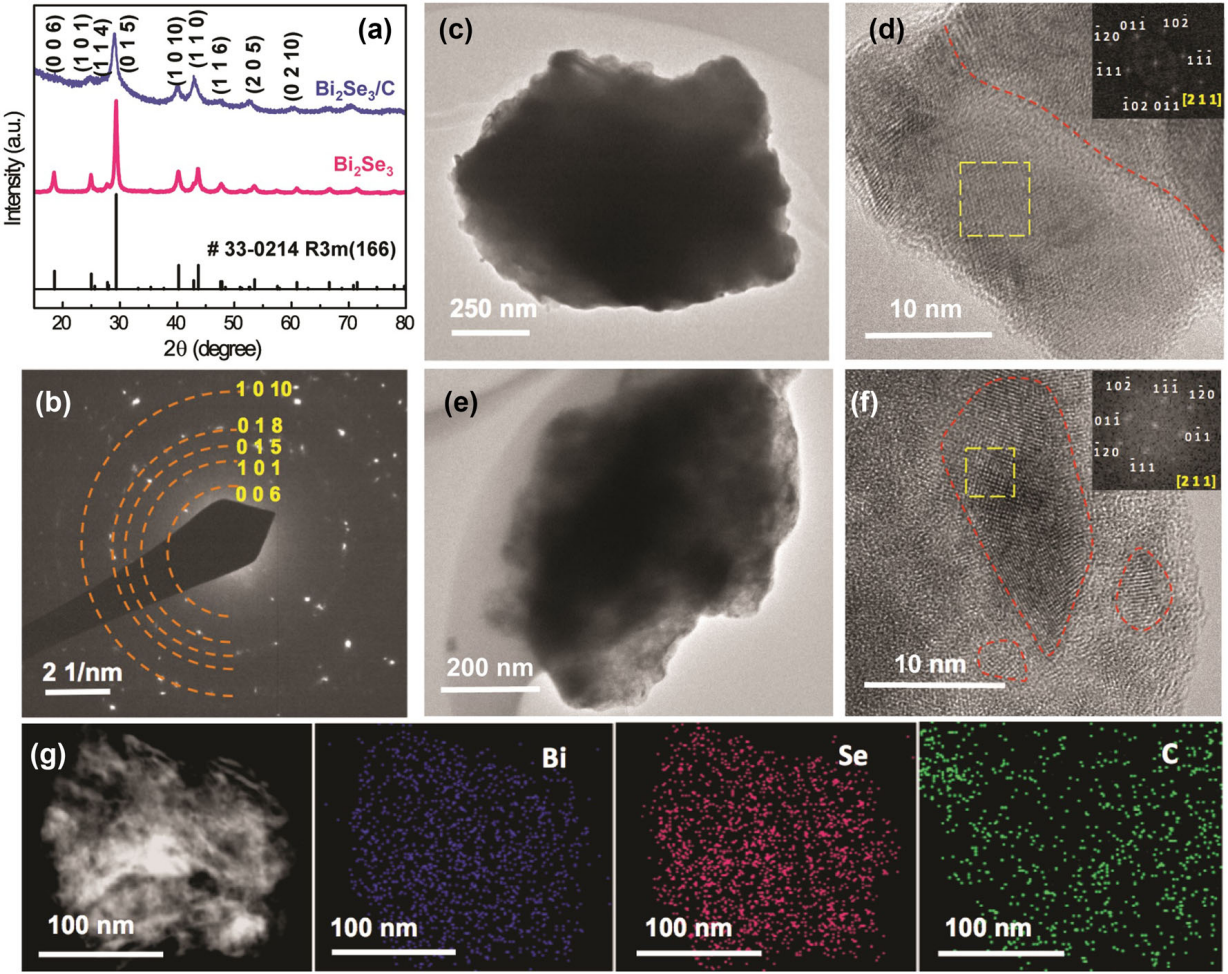
**a** XRD patterns of as-synthesized Bi_2_Se_3_ and Bi_2_Se_3_/C. **b** The diffraction pattern of Bi_2_Se_3_. **c** Low- and **d** high-resolution TEM images of Bi_2_Se_3_. **e** Low- and **f** high-resolution TEM images of Bi_2_Se_3_/C. **g** Scanning TEM (STEM) image and its corresponding elemental (Bi, Se, and C) mappings

Figure 1 displays the structural and morphological characterization details of Bi_2_Se_3_ and Bi_2_Se_3_/C. The XRD patterns of Bi_2_Se_3_ and Bi_2_Se_3_/C are shown in Fig. 1a. The ball milled Bi_2_Se_3_ and Bi_2_Se_3_/C display the same XRD patterns, which match well with the pure rhombohedral phase (space group $R\bar{3}m$ (166), JCPDS card No. 33-0214). After ball milling with carbon for another 6 h, the peaks of Bi_2_Se_3_/C become broader, indicating that smaller nanocrystals are produced. The crystal structure is again confirmed in the electron diffraction patterns of Fig. 1b, with the rings well indexed as the planes (0 0 6), (1 0 1), (0 1 5), (0 1 8), and (1 0 10) of rhombohedral-phase Bi_2_Se_3_. The TEM image in Fig. 1c and high-resolution TEM image in Fig. 1d show that the secondary Bi_2_Se_3_ particles are composed of well-developed nanocrystals with sizes ranging from a few nanometers to tens of nanometers. Figure 1e, f demonstrate that the Bi_2_Se_3_ nanocrystals are well encapsulated and uniformly distributed in the carbon matrix after integration with carbon. The primary nanocrystal sizes are approximately 5–20 nm, much smaller than those of as-synthesized Bi_2_Se_3_ because the carbon matrix can well separate and stabilize Bi_2_Se_3_ nanocrystals [32]. To reflect nanocrystal sizes across the samples, additional high-resolution TEM images are provided in Fig. S1. The particle sizes of the Bi_2_Se_3_/C nanocomposite also grow finer due to the separation of carbon compared to those of bare Bi_2_Se_3_, as observed in the SEM images (Fig. S2). Clear fringes of the crystal planes of Bi_2_Se_3_ can be found in Fig. 1f, indicating that the Bi_2_Se_3_ maintains good crystallinity in the carbon composite. In Fig. 1g, the uniform distribution of the elements Bi, Se, and C is confirmed by the EDS mapping. The carbon content of the composite is further confirmed to be 20.7 wt% by the TGA test (Fig. S3).

**Fig. 2:**
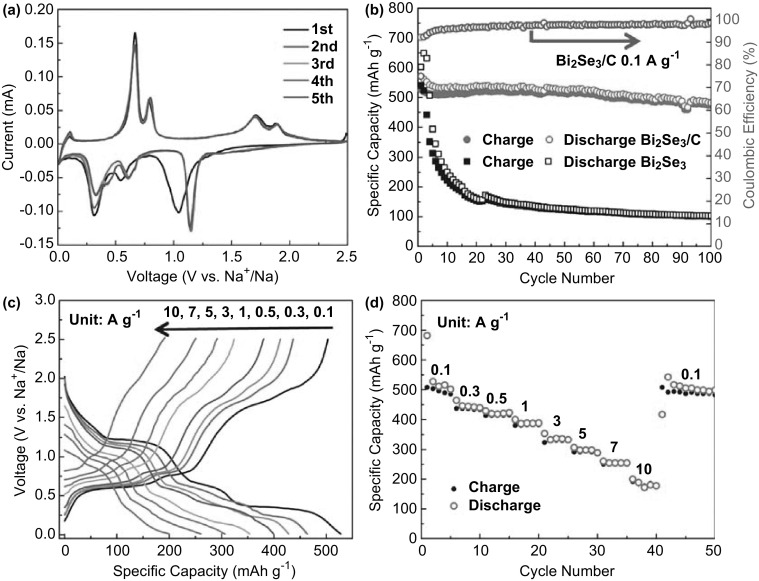
Studies of electrochemical properties of the Bi_2_Se_3_/C anode for SIBs. **a** CV curves of the Bi_2_Se_3_/C anode at 0.1 mV s^−1^. **b** Cyclic performance of Bi_2_Se_3_/C and Bi_2_Se_3_ anodes at 0.1 A g^−1^ and the related Coulombic efficiency of Bi_2_Se_3_/C anode. **c** Discharge/charge profiles and **d** rate performance of Bi_2_Se_3_/C anode at different current densities

The half-cell of Bi_2_Se_3_/C was cycled at a scan rate of 0.1 mV s^−1^ within 0.01–2.5 V versus Na^+^/Na and the *I*–*V* curves are shown in Fig. 2a. Three cathodic peaks at 1.04, 0.52, and 0.27 V and four anodic peaks at 1.88, 1.7, 0.79, and 0.67 V are depicted in the first cycle. The peak positions are analogous to those in Bi_2_S_3_ anode because of the similar properties between S and Se as chalcogens [14, 19, 20]. In the cathodic scan, Bi and Na_2_Se form at 1.04 V [19, 20], followed by the sodiation of Bi at lower voltages of 0.52 and 0.27 V [14]. In the reverse scan, desodiation of the Na–Bi alloy occurs at 0.67 and 0.79 V [14], then NaBiSe_2_ is formed at 1.7 and 1.88 V [19, 20, 35]. The peak at 1.04 V in the first cycle is slightly shifted to 1.14 V in the following cycle. Other than this shift, the CV curves overlap very well, which indicates a highly reversible Na storage kinetics. Figure S4 also displays the CV curve of Bi_2_Se_3_. The same characteristics are observed in the CV curves of Bi_2_Se_3_ and Bi_2_Se_3_/C, which indicate that integrating carbon does not affect the sodiation process of Bi_2_Se_3_. However, integrating carbon does improve the stability of the electrode, which is evidenced by the obvious decrease in the peak intensities of bare Bi_2_Se_3_ over CV cycling.

The cyclic performances of Bi_2_Se_3_ and Bi_2_Se_3_/C at 0.1 A g^−1^ and the related Coulombic efficiency of the Bi_2_Se_3_/C anode are shown in Fig. 2b. Alloying and conversion anodes often show lower Coulombic efficiencies than intercalation anodes. At the first cycle, the Bi_2_Se_3_ and Bi_2_Se_3_/C anodes both display reasonably high Coulombic efficiencies (> 75%), indicating higher utilization of Na^+^ than most alloying anodes. With carbon integrated, the reversible capacity of Bi_2_Se_3_/C anode (527 mAh g^−1^) is somewhat comprised compared to the capacity of 557 mAh g^−1^ for the Bi_2_Se_3_ anode at the first cycle. In the following cycles, however, the Bi_2_Se_3_/C anode exhibits much improved stability, reaching a steady value of 510 mAh g^−1^ within five cycles and retaining 89% of the initial capacity over 100 cycles, while the Bi_2_Se_3_ anode displays a fast decay in capacity to below 200 mAh g^−1^ within 20 cycles. At a higher current density of 0.5 A g^−1^, the Bi_2_Se_3_/C anode still shows high stability with an initial capacity of 445 mAh g^−1^ after the first two cycles at 0.1 A g^−1^ and that of 383 mAh g^−1^ over 180 cycles (Fig. S5). The cyclic performance of Bi_2_Se_3_/C is superior to those of other Bi-based materials and competitive with many typical anode materials (Table S1) [36–41]. Although the initial capacity of Bi_2_Se_3_/C is not extremely high compared to those of similar materials reported, the unique advantage of this composite is its stability in long-term cycling. For example, at 0.1 A g^−1^, the capacity of 470 mAh g^−1^ for Bi_2_Se_3_/C composite at the 100th cycle is more than triple that of Bi@C microspheres [42] and ca. 50% higher than that of Bi_2_S_3_ nanorods at the 40th cycle [19]. Figure 2c shows the voltage profiles of the Bi_2_Se_3_/C anode for a wide range of discharge/charge rates between 0.01 and 2.5 V versus Na^+^/Na. At the low current density of 0.1 A g^−1^, the plateaus can be clearly identified with three discharge plateaus and four charge plateaus, corresponding to the peaks in the CV curves. The discharge/charge profiles maintain analogous shapes and plateaus even at very high current densities, indicating the fast reaction kinetics of the Na storage process. The details of the fast reaction kinetics may be ascribed to the fast capacitive contribution, as discussed later. Figure 2d shows the excellent rate capability of Bi_2_Se_3_/C as an anode material for SIBs. Remarkably, it delivers the high capacities of 500, 445, 415, 384, 332, 298, 255, and 186 mAh g^−1^ at 0.1, 0.3, 0.5, 1, 3, 5, 7, and 10 A g^−1^, respectively. To confirm the high reversibility, 0.1 A g^−1^ is applied again after cycling at 10 A g^−1^, and the capacity returns to its previous level as expected. The rate capacities of Bi_2_Se_3_/C are competitive with those of typical anode materials listed in Table S2 and the performance is better at high current densities. The volumetric capacity is also an important consideration for practical application; that of the Bi_2_Se_3_/C electrode reaches 1064 mAh cm^−3^, calculated by multiplying the volumetric density of Bi_2_Se_3_/C (2.02 g cm^−3^) with the gravimetric capacity (527 mAh g^−1^) at 0.1 A g^−1^.

**Fig. 3:**
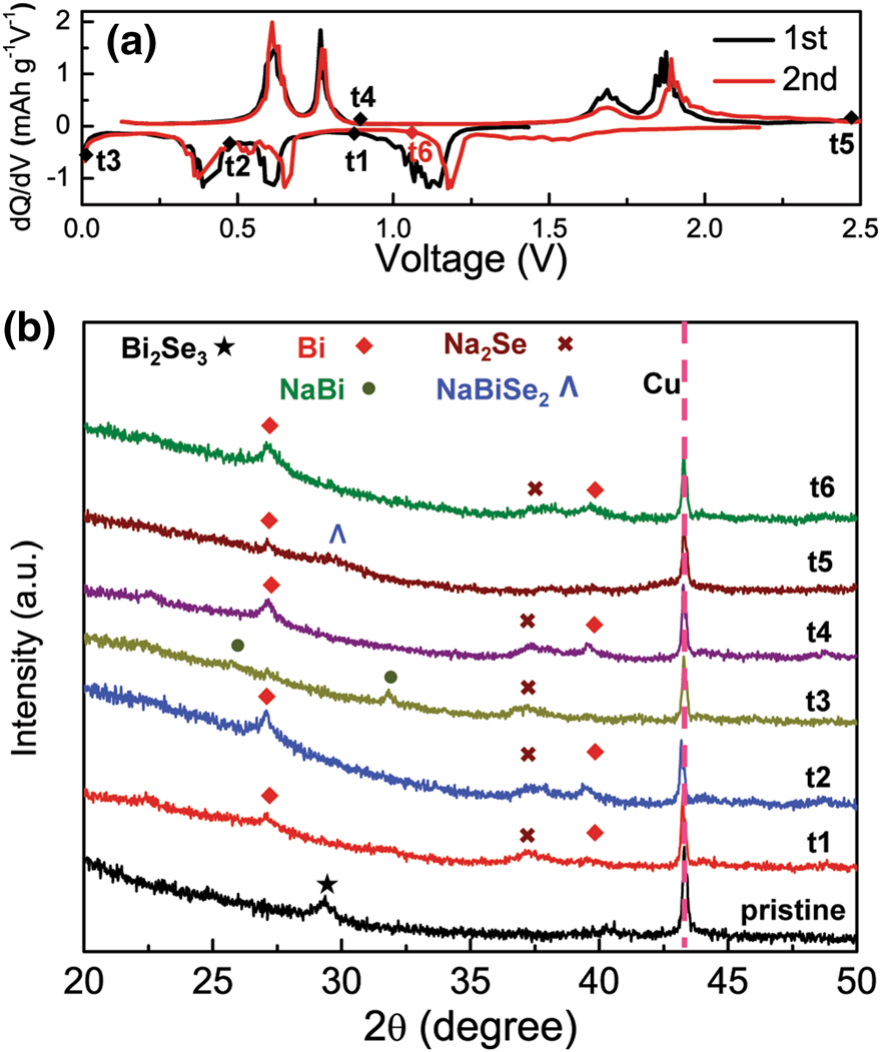
a d*Q*/d*V* plots for the first two cycles and **b** ex-situ XRD results of the Bi_2_Se_3_/C anode

To explore the insights of sodiation/desodiation mechanism of Bi_2_Se_3_, ex situ XRD was conducted. After charging/discharging, the electrodes were removed from the cells in a glove box and covered with Kapton tapes to avoid oxidation. The sampling points were chosen in reference to the d*Q*/d*V* curves in Fig. 3a. When the anode is sodiated to 0.86 V from the open-circuit voltage, the Bi_2_Se_3_ characteristic peak disappears while Bi peaks appear with Na_2_Se [14]. The XRD patterns of Bi and Na_2_Se are maintained when the material is discharged to a low voltage of 0.47 V. In this process, the intercalation of Na^+^ into Bi may occur. The phase of NaBi appears at 0.01 V, indicating that alloying reaction occurs at the complete sodiation state [8, 43]. In the desodiation process, Bi dealloys with Na^+^, evidenced by the appearance of the Bi phase at 0.88 V. However, even at the highest potential of 2.5 V, the Bi_2_Se_3_ phase does not recover; instead, NaBiSe_2_ with Bi phases are formed [19]. The irreversible Bi_2_Se_3_ change can also explain the peak shifting from 1.04 V in the first cycle to 1.14 V in the following cycles in the *I*–*V* curves of Fig. 2a. When the electrode is again sodiated to 1.05 V at the second cycle, diffraction patterns corresponding to Bi and Na_2_Se appear again. In summary, the phase changes during cycling can be listed as the following four steps:

Sodiation process:

1 $$ {\text{conversion}}\;{\text{reaction: Bi}}_{2} {\text{Se}}_{3} + \, 6{\text{Na}}^{ + } + \, 6{\text{e}}^{ - } \to 2{\text{Bi}} + 3{\text{Na}}_{2} {\text{Se }}\left( {\text{irreversible}} \right) $$

2 $$ {\text{alloying}}\;{\text{reaction}}{:}{\text{ Bi}} + x{\text{Na}}^{ + } + \, x{\text{e}}^{ - } \to {\text{Na}}_{x} {\text{Bi}} $$

Desodiation process

3 $$ {\text{dealloying}}\;{\text{reaction}}{:}{\text{ Na}}_{x} {\text{Bi}} \to {\text{Bi }} + \, x{\text{Na}}^{ + } + \, x{\text{e}}^{ - } $$

4 $$ {\text{conversion}}\;{\text{ reaction}}{:}{\text{ Bi}} + 2{\text{Na}}_{2} {\text{Se}} \to {\text{NaBiSe}}_{2} + 3{\text{Na}}^{ + } + \, 3{\text{e}}^{ - } $$

**Fig. 4:**
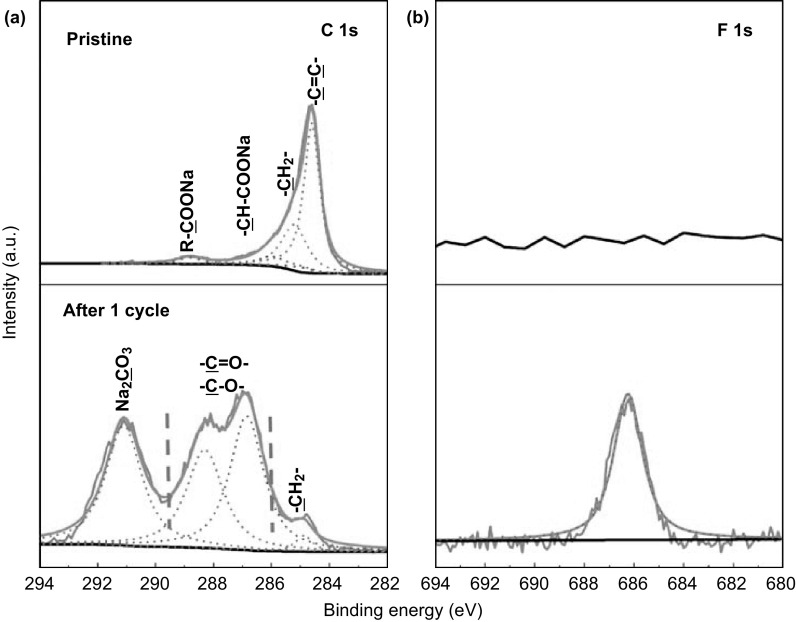
High-resolution XPS spectra **a** C 1*s* and **b** F 1*s* of the Bi_2_Se_3_/C electrode before and after one cycle

In addition to XRD analysis, XPS was also applied to provide a more comprehensive understanding of the materials and the electrochemical process, because XPS is sensitive to the surface within the depth of ca. 5 nm. Figure S7 displays the XPS survey spectrum and high-resolution spectra of Bi 4*f* and Se 3*d* for Bi_2_Se_3_/C electrode. The peaks at 163.7 and 158.4 eV are assigned to Bi 4*f*_5/2_ and Bi 4*f*_7/2_, respectively. The peaks at 54.3 and 53.5 eV correspond to Se 3*d*_3/2_ and Se 3*d*_5/2_ in Bi_2_Se_3_, respectively, confirming the successful synthesis of Bi_2_Se_3_ [44]. In addition, the peaks related to BiO_*x*_ and SeO_*x*_ are also found, indicating oxidation happens at the surface [44]. The solid electrolyte interface (SEI) compositions were also investigated by comparing the electrode before and after one cycle. Figure 4 indicates significant changes in the C 1*s* and F 1*s* spectra. The pristine electrode has a strong signal at 284.6 eV related to the carbon bonds of graphite or carbon black, and the small peaks at 285.3, 285.9, and 288.8 eV correspond to –CH_2_–, –CH–COONa, and R–COONa of the PAA binder [9, 45]. After one cycle, several new peaks are formed in the higher binding energy region and the strong signal at 284.6 eV related to graphite and carbon black disappears, indicating the formation of the SEI film on the surface. The signals from 286.0 to 289.5 eV are assigned to the –C–O– and –C=O– species of the SEI film and the peak at 291.1 eV arises from Na_2_CO_3_ of the SEI film [9, 46]. The signal related to F 1*s* appears after one cycle, indicating that the SEI film contains F from the decomposition of FEC.

**Fig. 5:**
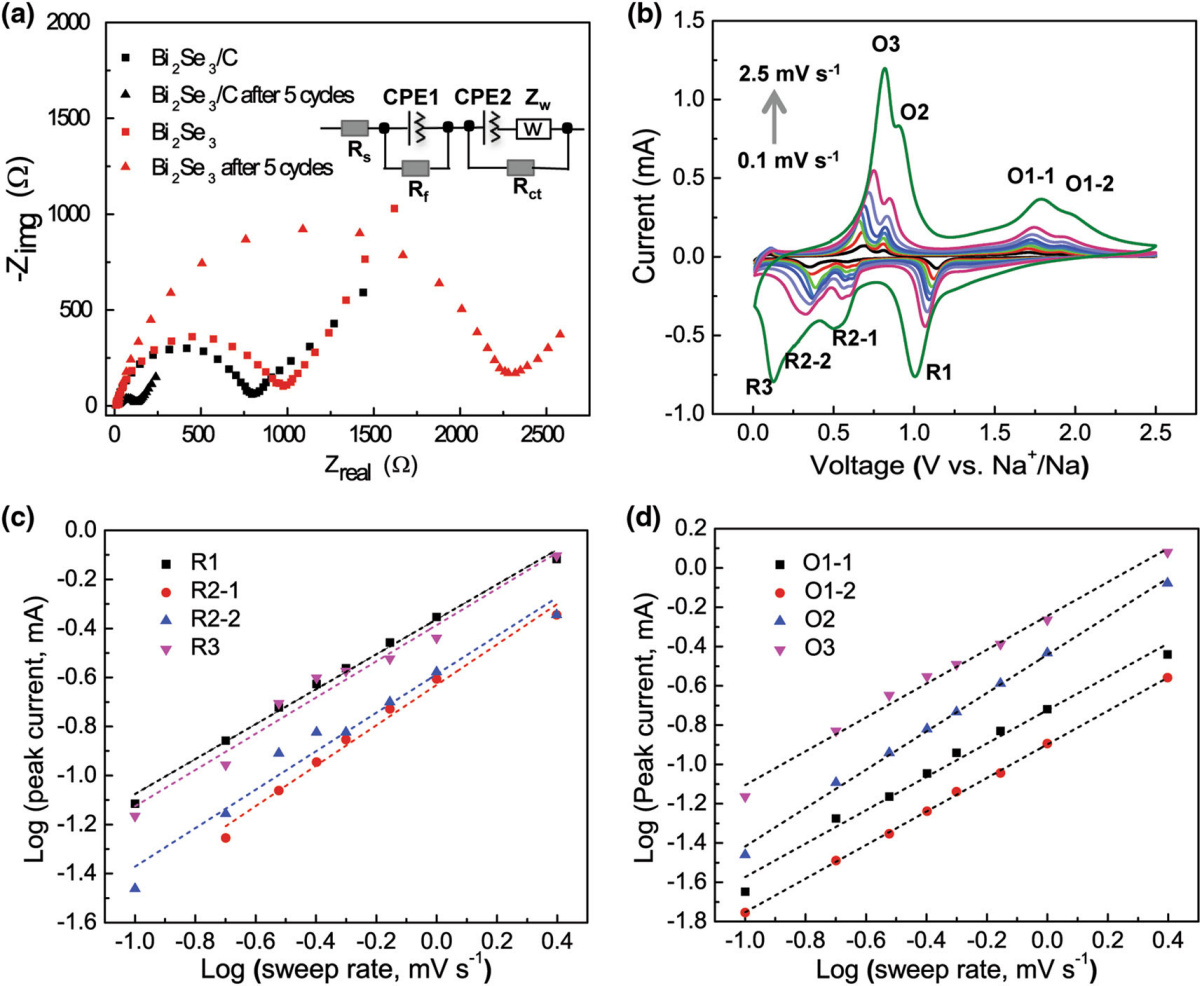
**a** EIS spectra for Bi_2_Se_3_ and Bi_2_Se_3_/C anodes before and after five cycles. **b** CV curves of the Bi_2_Se_3_/C anode at different scan rates. **c, d** *b*-value fitted by the relationship of the logarithm peak currents and logarithm scan rates

The reaction kinetics can be revealed by EIS and the EIS spectra of Bi_2_Se_3_/C electrode and Bi_2_Se_3_ electrode are displayed in Fig. 5a. The intercept with the *Z*_real_ axis at high frequency represents the electrolyte and contact resistance (*R*_s_), while the semicircles at medium frequency are related to the SEI resistance (*R*_f_) and electrolyte/electrode charge transfer resistance (*R*_ct_) [47]. The equivalent circuit model for the fitting is shown in the inset of Fig. 5a with the fitting results listed in Table S3. *R*_ct_ of the Bi_2_Se_3_/C electrode decreases significantly from 661.4 to 81.4 Ω after cycling benefited from the reconstructed porous structure with close connections, as seen under SEM (Fig. S8) [48]. On the contrary, the EIS spectra of Bi_2_Se_3_ display a large *R*_ct_ increase after cycling due to the contact loss. For the Bi_2_Se_3_ electrode after cycling, large aggregates are formed with rough surfaces and loose contact between particles. In addition, the *R*_f_ increase of 19.9 Ω for the Bi_2_Se_3_ electrode is more significant than that of 6.8 Ω for the Bi_2_Se_3_/C electrode, caused by the fracture and the continuous growth of a thick SEI layer in the Bi_2_Se_3_ electrode.

For nanomaterials with large surface areas, surface-induced capacitive processes can have significant effects and improve the charge/discharge capability [49–51]. The *b* value is often used as an index to estimate the surface-induced capacitive contribution. According to *i *= a*ν*^*b*^, where *i* is the current response at the scan rate *ν*, the *b* value can be readily fitted by log(*i*) − log(*ν*) linear plots. The *b* value can vary from 0.5 to 1. The capacitive process dominates when the *b* value is close to 1, while diffusion-controlled processes dominate when the *b* value approaches 0.5. Figure 5b shows the *I*–*V* curves at different scan rates for the Bi_2_Se_3_/C electrode; the relations of log(*i*) and log(*ν*) at the corresponding peaks derived from the *I*–*V* curves are shown in Fig. 5c, d. The fitted *b* values are 0.71, 0.82, 0.78, and 0.74 for the R_1_, R_2–1_, R_2–2_, R_3_ peaks and 0.85, 0.85, 0.98, and 0.86 for O_1–1_, O_1–2_, O_2_, and O_3_ peaks. These values are much higher than 0.5, which indicates that fast capacitive process occurs during Na storage, contributing to the high rate capacity for the Bi_2_Se_3_/C electrode. The current and scan rate relations are not shown for Bi_2_Se_3_ electrode because of the significant changes of the CV curves over cycling.

## Conclusions

The application of Bi_2_Se_3_ was explored as an anode material for SIBs. Benefiting from the high theoretical capacity and high intrinsic conductivity of Bi_2_Se_3_, the positive effects of carbon, and the effective HEBM method, a high-performance anode material was achieved. The Bi_2_Se_3_/C electrode showed a high reversible capacity of 527 mAh g^−1^ and retains 89% of this capacity over 100 cycles at 0.1 A g^−1^. To obtain insights regarding the electrochemical process of Na storage, the phase changes were revealed by ex situ XRD.

## Electronic supplementary material

Below is the link to the electronic supplementary material.

Supplementary material 1 (PDF 828 kb)
