# Supplementary material for: Bi2Se3/C Nanocomposite as a New Sodium-Ion Battery Anode Material
Source: Nanomicro Lett. 2018 May 3;10(3):50. doi: 10.1007/s40820-018-0201-9 (PMC6199094; doi:10.1007/s40820-018-0201-9)
Supplement: Supplementary file 1 — Supplementary material 1 (PDF 828 kb) [file 40820_2018_201_MOESM1_ESM.pdf]

Supporting Information for

Bi_2Se_3/C Nanocomposite as a New Sodium-Ion Battery Anode Material

Lixin Xie[1], Ze Yang[1], Jingying Sun[1], Haiqing Zhou[1], Xiaowei Chi[2], Hailong Chen[3], Andy X. Li[4], Yan Yao[2], Shuo Chen[1,*]

[1]Department of Physics and TcSUH, University of Houston, Houston, TX 77204, USA

[2]Department of Electrical and Computer Engineering and Materials Science and Engineering Program, University of Houston, Houston, TX 77204, USA

[3]The Woodruff School of Mechanical Engineering, Georgia Institute of Technology, Atlanta, Georgia 30332, USA

[4]Clements High School, 4200 Elkins Dr, Sugar Land, TX 77479, USA

*Corresponding author. Email: schen34@uh.edu (Shuo Chen)

Supplementary Figures and Tables

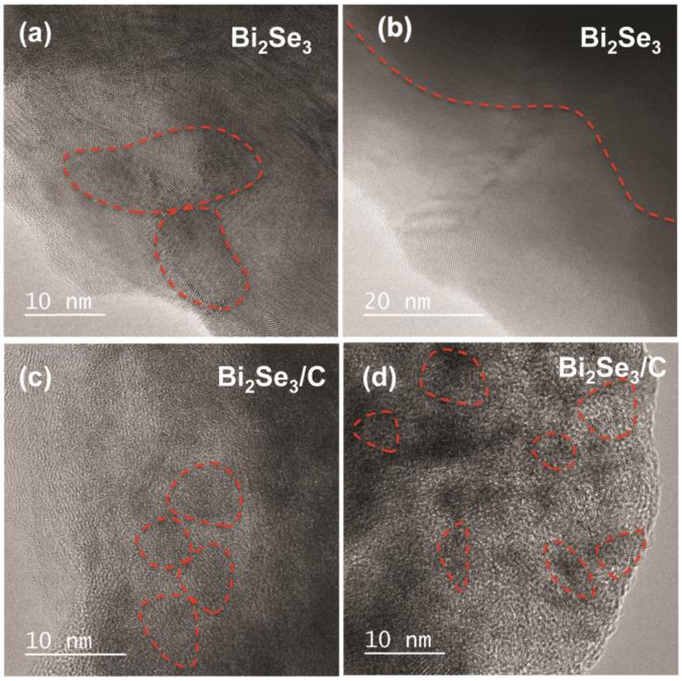

Fig. S1 HR-TEM images of **a-b** Bi_2Se_3 and **c-d** Bi_2Se_3/C. The nanocrystals are marked by red lines

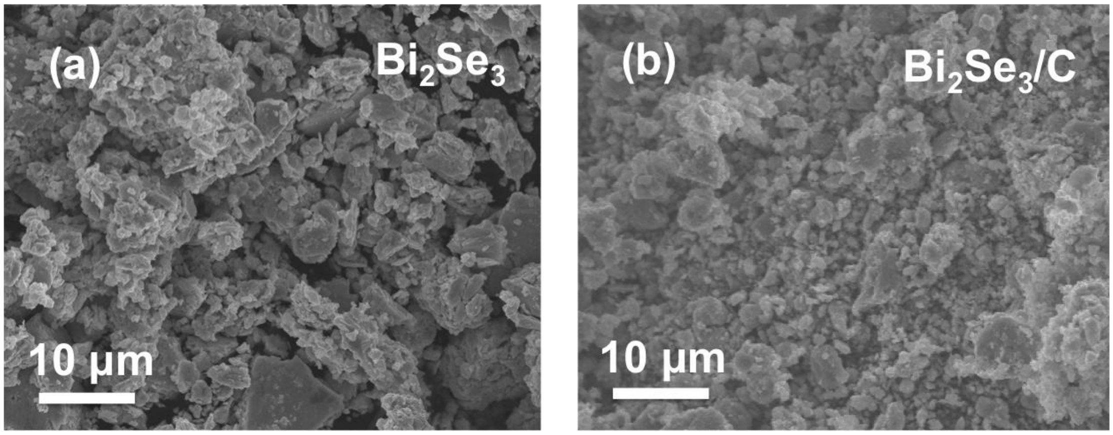

Fig. S2 SEM images of as-synthesized **a** Bi_2Se_3 and **b** Bi_2Se_3/C

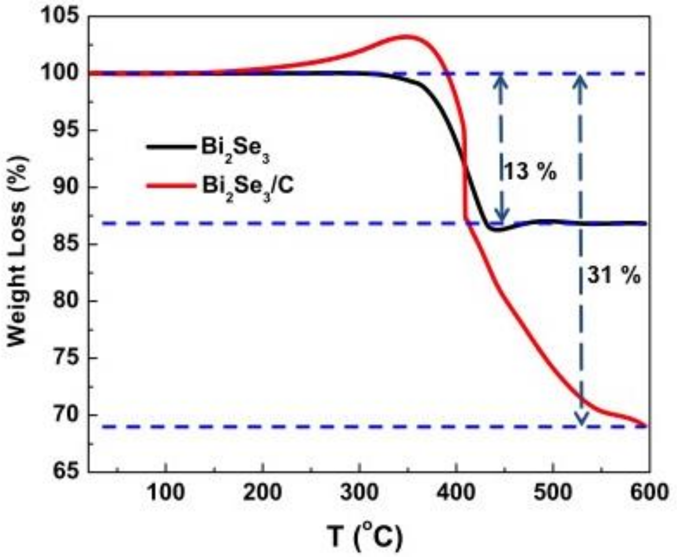

Fig. S3 Thermogravimetric analysis of Bi_2Se_3 and Bi_2Se_3/C

At temperature higher than 330 °C, the decomposition of Bi_2Se_3 leads to a weight loss of 13 wt%. Bi_2Se_3/C first experiences a weight increase due to the oxidation of carbon starting from 200 °C and finally a sudden drop from 400 to 600 °C indicating the total burn down of carbon. In 1 mg Bi_2Se_3/C composite, we suppose the mass of Bi_2Se_3 to be x mg and carbon to be (1-x) mg. The carbon is burnt out completely in TGA measurement of Bi_2Se_3/C. The relation can be listed as following:

$$0.13x + (1-x) = 0.31$$

By cracking x, the carbon content is determined to be 20.7 wt%.

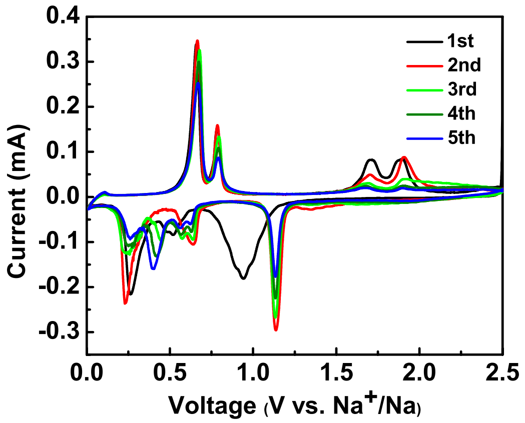

Fig. S4 CV curves of Bi_2Se_3 anode at a scan rate of 0.1 mV s^{-1}

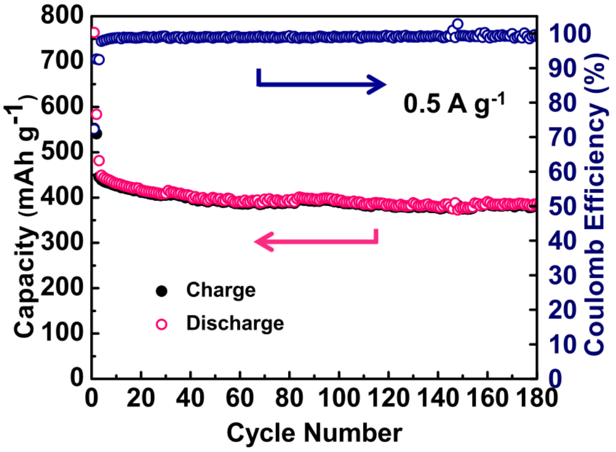

Fig. S5 The cyclic performance of Bi_2Se_3/C anode at the current density of 0.5 A g^{-1}. The first two cycles were performed under a low current density of 0.1 A g^{-1}

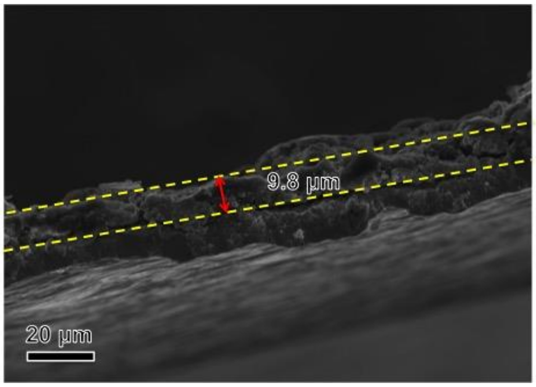

Fig. S6 SEM image of the electrode film at the cross section

The film on the Cu foil is determined to ca. 9.8 μm under SEM. On the Cu foil with an area of 0.504 cm^2, the total loading of material is 1 mg including the active material Bi_2Se_3/C, carbon black and binder. Thus the volumetric density is calculated to be 2.02 g cm^{-3}. The volumetric capacity is calculated by multiplying the volumetric density of Bi_2Se_3/C (2.02 g cm^{-3}) with the gravimetric capacity.

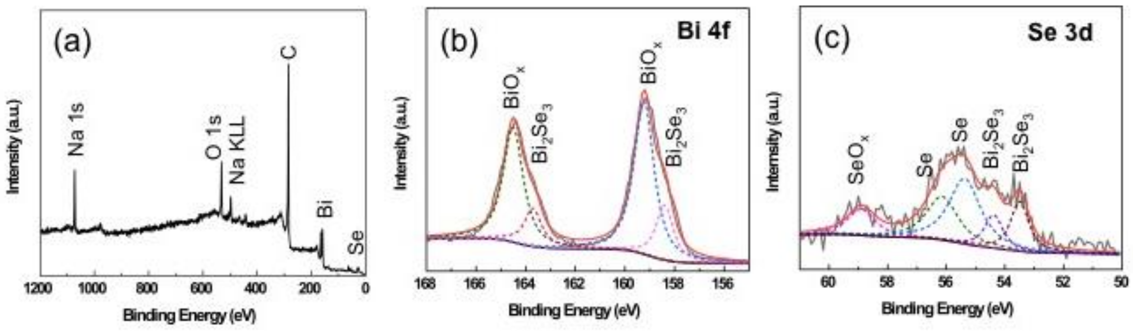

Fig. S7 X-ray photoelectron spectroscopy of pristine Bi_2Se_3/C electrode. **a** survey spectrum; high-resolution spectra for **b** Bi 4f and **c** Se 3d

The possible reason for the existence of element Se is that the precipitation of Se out of Bi_2Se_3 crystal is thermodynamically favorable.

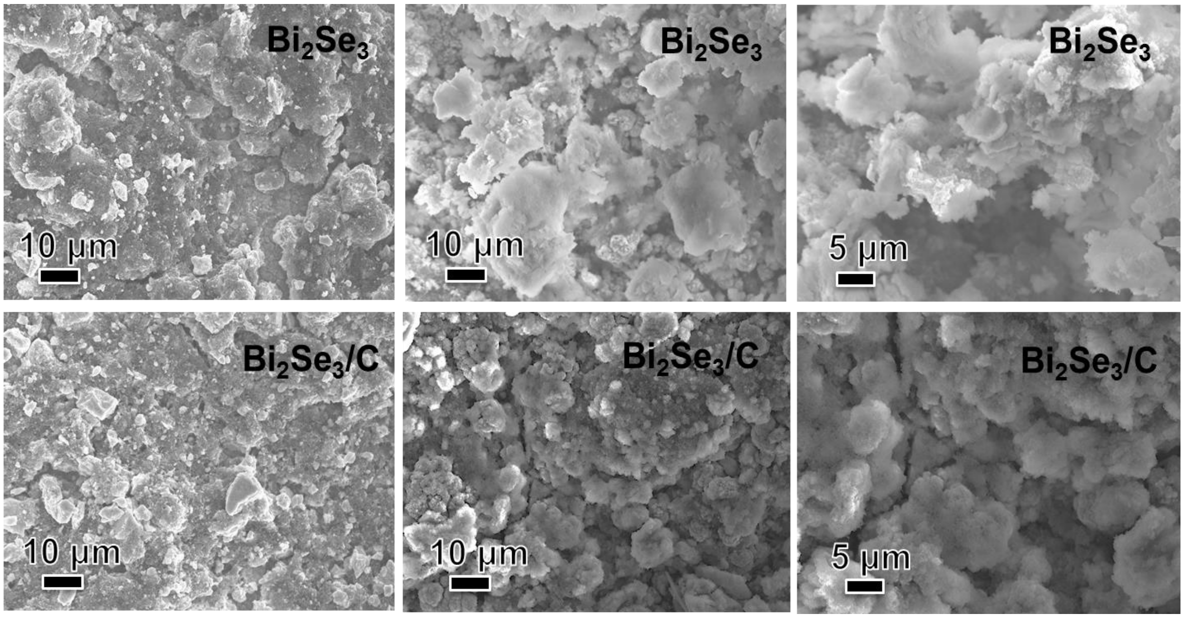

Fig. S8 SEM images of the Bi_2Se_3 electrode and Bi_2Se_3/C electrode. **a** pristine Bi_2Se_3 electrode and **d** Bi_2Se_3/C electrode; **b-c** Bi_2Se_3 electrode and **e-f** Bi_2Se_3/C electrode after 20 cycles at different magnification scale

Table S1 Electrochemical cyclic performance comparison of this work versus the reported Bi-based materials and other typical anode materials for SIBs

Samples	Initial capacity (mAh g^{-1})	Cyclic stability[1] (mAh g^{-1})	Current density (A g^{-1})	Voltage range (V)	References
Bi_2Se_3/C nanocomposite	527	470 (100)	0.1	0.1–2.5	This work
Bi_2Se_3/C nanocomposite	445	382 (180)	0.5	0.1–2.5	This work
Bi@graphene	358	203 (50)	0.04	0.3–0.9	[1]
Bi nanorod bundles	367	302 (150)	0.05	0.1–0.9	[2]
Bi@C microsphere	299	123.5 (100)	0.1	0.01–2	[3]
Bi_2S_3 nanorods	658	322 (40)	0.1	0.05–3	[4]
Bi_2S_3 @ CNT nanocomposite	795	85 (60)	0.06	0.01–3	[5]
Carbon-capsulated F_3O_4	657	513 (60)	0.1	0.005–3	[6]
Carbon-capsulated Co_3O_4	583		0.1	0.005–3	[6]
$NiCo_2O_4$ hollow spheres	513	341 (100)	0.1	0.01–3	[7]
NC @ SnO_2	212.5	270 (100)	0.1	0.001–3	[8]
MoO_2 @ NC nanofibers	372	350 (200)	0.1	0.001–3	[9]
ReS_2/N-CNFs	456	245 (800)	0.1	0.01–3	[10]
FeS_2/rGO	759.4	581.7 (100)	0.1	0.1–2.3	[11]

[1]The number in the bracket indicates the cycle number.

Table S2 Rate capacity comparison of this work versus the reported Bi-based materials other typical anode materials for SIBs (Unit: mAh g^{-1})

Samples	Current density (A g^{-1})								References
	0.1	0.2	0.3	0.5	1	2	3	5	
Bi_2Se_3/C nanocomposite	500		445	415	384		332	298	This work
Bi nanorod bundles	292			212	143	102			[2]
Bi@C microsphere	299	252		192	141	90			[3]
Bi_2S_3 nanorods		502	444	391	332	264			[4]
Bi_2S_3@CNT nanocomposite			50				25		[5]
Carbon-capsulated F_3O_4		510		425	330	246		163	[6]
Carbon-capsulated Co_3O_4		583		416	310	251		183	[6]
$NiCo_2O_4$ hollow spheres	511	412		353	251				[7]
NC@SnO_2	300	280			193				[8]
MoO_2@NC nanofibers	372		348	329	299	260			[9]
FeS_2/rGO	705	672		613	555	496	426		[11]

Table S3 Comparison of the impedances of electrodes before and after 5 cycles from EIS results

Samples	R_s (Ω)	R_{ct} (Ω)	R_f (Ω)
Bi_2Se_3/C pristine	10.5	661.4	10.5
Bi_2Se_3/C after cycling	11.7	81.4	17.3
Bi_2Se_3 pristine	8.6	777.2	46.6
Bi_2Se_3 after cycling	17.4	2004.0	66.5

References

[1] D. Su, S. Dou, GuoxiuWang, Bismuth: A new anode for the Na-ion battery. Nano Energy **12**, 88-95 (2015). https://doi.org/10.1016/j.nanoen.2014.12.012

[2] S. Liu, J. Feng, X. Bian, J. Liu, H. Xu, Advanced arrayed bismuth nanorod bundle anode for sodium-ion batteries. J. Mater. Chem. A **4**(26), 10098-10104 (2016). https://doi.org/10.1039/c6ta02796b

[3] F. Yang, F. Yu, Z. Zhang, K. Zhang, Y. Lai, J. Li, Bismuth nanoparticles embedded in carbon spheres as anode materials for sodium/lithium-ion batteries. Chem. Eur. J. **22**(7), 2333-2338 (2016). https://doi.org/10.1002/chem.201503272

[4] W. Sun, X. Rui, D. Zhang, Y. Jiang, Z. Sun, H. Liu, S. Dou, Bismuth sulfide: A high-capacity anode for sodium-ion batteries. J. Power Sources **309**, 135-140 (2016). https://doi.org/10.1016/j.jpowsour.2016.01.092

[5] W. Yang, H. Wang, T. Liu, L. Gao, A Bi_2S_3@CNT nanocomposite as anode material for sodium ion batteries. Mater. Lett. **167**, 102-105 (2016). https://doi.org/10.1016/j.matlet.2015.12.108

[6] Y. Zhou, W. Sun, X. Rui, Y. Zhou, W.J. Ng, Q. Yan, E. Fong, Biochemistry-derived porous carbon-encapsulated metal oxide nanocrystals for enhanced sodium storage. Nano Energy **21**, 71-79 (2016). https://doi.org/10.1016/j.nanoen.2015.12.003

[7] X. Zhang, Y. Zhou, B. Luo, H. Zhu, W. Chu, K. Huang, Microwave-assisted synthesis of $NiCo_2O_4$ double-shelled hollow spheres for high-performance sodium Ion batteries. Nano-Micro Lett. **10**(1), 13 (2017). https://doi.org/10.1007/s40820-017-0164-2

[8] [8] J. Liang, C. Yuan, H. Li, K. Fan, Z. Wei, H. Sun, J. Ma, Growth of SnO_2 nanoflowers on N-doped carbon nanofibers as anode for Li- and Na-ion batteries. Nano-Micro Lett. **10**(2), 21 (2018). https://doi.org/10.1007/s40820-017-0172-2

[9] J. Liang, X. Gao, J. Guo, C. Chen, K. Fan, J. Ma, Electrospun MoO_2@NC nanofibers with excellent Li^+/Na^+ storage for dual applications. Sci. China Mater. **61**(1), 30-38 (2017). https://doi.org/10.1007/s40843-017-9119-2

[10] M. Mao, C. Cui, M. Wu, M. Zhang, T. Gao, X. Fan, J. Chen, T. Wang, J. Ma, C. Wang, Flexible ReS_2 nanosheets/ N-doped carbon nanofibers-based paper as a universal anode for alkali (Li, Na, K) ion battery. Nano Energy **45**, 346-352 (2018). https://doi.org/10.1016/j.nanoen.2018.01.001

[11] Q. Wang, C. Guo, Y. Zhu, J. He, H. Wang, Reduced graphene oxide-wrapped FeS_2 composite as anode for high-performance sodium-ion batteries. Nano-Micro Lett. **10**, 30 (2018). https://doi.org/10.1007/s40820-017-0183-z
